# 肺小结节术前CT引导下Hook-wire定位的临床应用

**DOI:** 10.3779/j.issn.1009-3419.2011.05.07

**Published:** 2011-05-20

**Authors:** 夏轶 闾, 运海 杨, 坚 胡, 一鸣 倪

**Affiliations:** 310003 杭州，浙江大学医学院附属第一医院胸心外科 Department of Cardiothoracic Surgery, the First Afliated Hospital of Medical School of Zhejiang University, Hangzhou 310003, China

**Keywords:** CT引导, Hook-wire, 肺结节, CT-guided, Hook-wire, Pulmonary nodule

## Abstract

**背景与目的:**

胸腔镜术中对于直径 < 1 cm的肺小结节较难准确定位。数年前即有学者开始尝试使用各种方法进行术前的肺小结节定位。本文从适应症、结果、并发症三个方面回顾性分析了胸腔镜术前CT引导下Hook-wire定位的临床应用价值。

**方法:**

2010年1月-2010年4月，20例患者于胸腔镜术前接受了CT引导下肺小结节Hook-wire定位。小结节直径从0.5 cm-2 cm（平均9.8 cm±5.3 cm）。评价指标包括定位成功率，定位相关并发症，中转开胸比率等。

**结果:**

20例患者中18例定位成功，CT定位花费时间平均为14.5 min，全组无严重并发症发生。

**结论:**

胸腔镜术前CT引导下肺小结节Hook-wire定位有一定的临床应用价值，可帮助术中精确定位肺小结节位置，并且并发症发生率较低。

## Abstract (en)

**Background and objective:**

It's difficult to localize the accurate position for some pulmonary nodules in video-assisted thoracoscopic surgery (VATS) wedge resection. The aim of this study is to retrospectively analyze the clinical significance of CT-guided preoperative pulmonary nodule localization technique.

**Methods:**

Between Jan 2010 and Apr 2011, 20 patients of the First Affiliated Hospital of Medical School of Zhejiang University underwent preoperative pulmonary nodule localization technique before performing VATS wedge resection of the pulmonary nodule. Diameter of the lesion ranges from 0.5 cm to 2 cm (average 9.8 cm±5.3 cm). It was evaluated with the success rate in localization technique, rate of localization related complications, and rate of transferring thoracotomy.

**Results:**

Eighteen patients underwent successful CT-guided Hookwire localization, with the average time of 14.5 minutes. There was no serious complications.

**Conclusion:**

CT-guided preoperative pulmonary nodule localization is a promising technique for small solitary pulmonary nodules. It could play an important role in accurate localization of small pulmonary nodules, and it is a safe technique with less postoperative complications.

由于近些年在健康体检和肿瘤患者随访中CT的广泛应用，肺部小结节越来越多的被发现。在临床工作中，由于近50%的肺小结节为恶性，有必要采取有创的手段获取病理学依据。CT引导下肺小结节活检和胸腔镜下肺楔形切除术是获取肺小结节组织学依据的标准手段。但对于直径 < 1.5 cm的肺小结节，穿刺活检的准确率明显下降。因此对于直径 < 1 cm的肺小结节，胸腔镜便成了获取组织学依据的唯一选择^[1]^。而对于胸腔镜手术，直径 < 1cm或胸膜下深度超过0.5 cm的小结节术中手指定位难度较大，而部分肿块本身质地较软，更增加了手术难度。一些胸腔镜手术因此不得不中转开胸。为此数年前即有学者开始尝试使用各种方法进行术前的肺小结节定位^[2]^。术前CT引导下的Hook-wire定位并不是新技术，但在国内开展尚不普遍，本文对近期浙江大学医学院附属第一医院胸心外科开展此项技术的20例患者做一回顾性分析。

## 1 材料与方法

### 1.1 设备与材料

① Hook-wire：德国宝雅医疗（Pajunk GmbH Mediziintechnologie）生产的乳腺穿刺定位针，产品规格20 G×100 mm，产品编号275S090120，导丝带加强。②CT：西门子大口径CT机，型号：Somaris/5 syngo CT 2006A。③激光定位：LAP LASER MODEL：Dorado-CT-4-1-Wall。

### 1.2 临床资料

回顾性分析自2010年1月-2010年4月接受术前CT引导下定位的20例患者。其中男8例，女12例，平均年龄50.5岁（范围18岁-71岁）。所有患者在术前评估存在胸腔镜下楔形切除可能者纳入研究（肿瘤距肺脏层胸膜距离 < 3.5 cm）。4例患者术前有恶性肿瘤病史，包括3例肝癌，1例软组织肉瘤。所有患者均只有1个肺小结节。全组病例的术前CT引导下穿刺均由同一医生完成。记录肺小结节的直径，距胸膜的距离，穿刺操作的时间（从局麻开始至平皮肤剪断Hook-wire）。记录患者最终病理结果，定位成功率，转开胸比率，胸腔镜手术时间，术后并发症，术中意外发生情况。

### 1.3 定位及手术方法

手术当日，患者于术前2 h送至CT室，患者及其家属签署知情同意。患者体位依穿刺所需最短路径而定，利用CT扫描仪特有的穿刺活检扫描序列，结合立体三维重建，利用激光定位线和体表标记明确穿刺点和进针通道。局部消毒局麻后，在CT引导下尽可能将穿刺针穿过小结节，若结节过小存在困难则将穿刺针穿过与结节尽可能相近的肺组织。套针位置满意后则释放Hook-wire，撤出套针，随即复查CT，确认Hookwire位置。若位置满意则平皮肤剪断金属线，患者送手术室手术。手术使用一次性切割闭合器楔形切除肿块，标本送冰冻。若病理为原发肺非小细胞肺癌则继续在全腔镜下行肺叶切除+淋巴结清扫术。

将患者小结节病理结果、定位成功率、胸腔镜中转开胸率、楔形切除手术时间及术后并发症进行分析。

## 2 结果

### 2.1 一般资料

CT引导下穿刺耗时平均为14.5 min（9 min-21 min），有8例患者定位针穿过结节，12例患者因肺部结节过小定位针穿过结节旁。2例患者患侧呼吸音降低，CT提示少量气胸，无需处理。2例患者穿刺后诉穿刺部位疼痛，疼痛评分4分，并于肺楔形切除术后缓解。全组无出血、无严重并发症发生。穿刺结束至手术开始时间间隔平均为1.5 h（0.5 h-2.2 h）。

### 2.2 定位失败

所有患者中，有2例定位失败，定位成功率90%。1例患者肺结节直径0.5 cm，位于斜裂边缘，穿刺针置于斜裂中，未穿过肺组织。该病例楔形切除通过手指触诊导引下切除。1例患者穿刺后体表钢丝未剪断，小结节位于肺脏层胸膜下，位置表浅，肺塌陷后胸腔镜探查发现钢丝已滑脱至胸腔内。该病例通过观察肺表面发现有穿刺创面轻微出血及气体逸出，得以定位肿块具体位置，亦在全腔镜下完成肺楔形切除术。

### 2.3 术中意外

术中意外2例。2例患者因结节位置较深，造成定位钢丝位置过深，其中1例在切除时定位钢丝被切割闭合器切断，残留在健肺中，随即在腔镜下行切开取出。另1例钢丝被切割闭合器刀头推开变形（图 1）。

**1:**
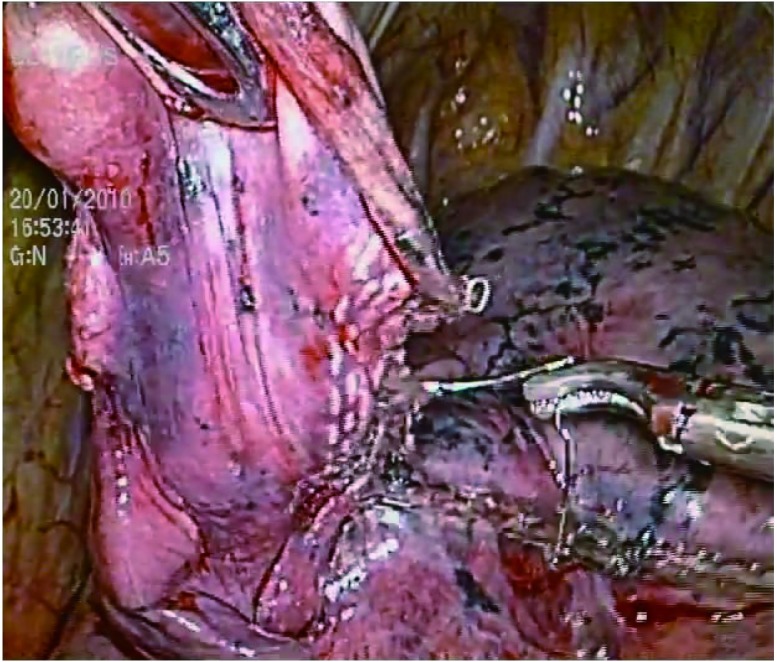
钢丝被切割闭合器刀头推开变形 Hook-wire was pushed out by the stapler

### 2.4 手术时间

肺楔形切除平均时间为45 min（20 min-95 min）。其中最长时间是由于钢丝被切割闭合器切断，残留在健肺中，在腔镜下行切开取出所致。病理结果提示肺原发癌10例，转移癌4例，良性病变6例。

## 3 讨论

近些年来随着微创胸外科的发展，胸腔镜手术日益为广大患者和医护人员接受，但对于1 cm以下、距脏层胸膜较深及肿块本身质地软的小结节，胸腔镜难以准确定位，从而导致一定比率的中转开胸和漏诊。为解决这一问题，临床上应用了很多方法予以术前或术中定位，如亚甲蓝注射、三维成像导航、术中超声定位、核素粒子注射定位等。每种方法都有其先进性和缺点。例如亚甲蓝注射可能会导致胸腔内及胸膜染料着色，从而使术者很难辨认具体的病灶位置。超声定位对超声操作者的依赖度高，而且部分肺气肿患者较难做到肺的完全塌陷。核素粒子则存在辐射危害等^[3]^。

CT引导下Hook-wire定位有其独特的优势：①CT引导下的定位过程简单快捷，平均耗时40 min；②有效减少VATS探查时间，缩短总手术时长；③Hook-wire可用于术中提拉肺组织，便于结节的切除^[4]^。

对于直径 < 1 cm，距脏层胸膜深度 > 0.5 cm的小结节，术中较难触及，适于术前定位。此外，肺部CT显示结节为毛玻璃样改变或提示密度不高者，以及位于术中手指难以触及的某些部位的肺小结节，即便直径 > 1 cm，术者对于肿块的具体位置、边界也很难明确，这种情况下CT引导下Hook-wire定位有很高的价值。

CT引导下Hook-wire定位的并发症发生率很低，本研究中2例患者出现无症状的轻微气胸，2例患者穿刺后诉穿刺部位疼痛，疼痛评分4分，应为Hook-wire留置刺激所致，肺楔形切除术后Hook-wire移除，疼痛自然缓解。

本组研究中有2例定位失败。1例患者肺结节直径0.5 cm，位于斜裂边缘，穿刺针置于斜裂中，未穿过肺组织。故对于叶裂边缘的微小肺结节，由于直接穿过结节存在困难，应避开叶裂，穿过旁边结节所在的肺叶组织。1例患者穿刺后体表钢丝未剪断，小结节位于肺脏层胸膜下，位置表浅，肺塌陷后胸腔镜探查发现钢丝已滑脱至胸腔内。故对于位置很表浅的微小肺结节，穿刺深度应达到2 cm，使得Hook-wire的整个倒钩结构埋于肺组织中，此外钢丝还应平皮肤剪断，可减少脱钩的发生。

本组研究中还有2例钢丝定位过深引起的切割闭合器误切钢丝发生，这两例患者肺结节深度均超过2 cm。钢丝定位深度则超过结节，最深达到3 cm。进行胸腔镜下肺楔形切除时，对于某些特定位置的肺结节，由于操作孔位置选择的关系，有时无法做到传统意义上的“楔”形切除，更多时候是梯形或钝三角形切除。另外，肺的一些钝边部位，很难进行较深的楔形切除，因此，对于深度超过2 cm，位于肺钝边（如下叶背段），直径 > 1 cm的肺部结节即便进行了Hook-wire定位，胸腔镜术中行楔形切除还是会面临很多风险，如：切缘阳性，过多肺组织被切除，切缘距离结节的合理距离难以保障，切断钢丝造成异物存留等^[5]^所以我们认为，对于位置很深的肺结节选择楔形切除要慎重，要有肺段或肺叶切除的准备。

由于存在不全切除可能以及脱钩风险，所有小结节尤其是毛玻璃样改变，质地柔软无法触及的小结节术前均应要求放射科行高分辨率薄层CT扫描明确小结节所在肺段，以备定位失败或异物存留时行肺段切除，消除医疗隐患。

综上所述，胸腔镜术前CT引导下Hook-wire定位操作快捷，穿刺定位准确有效，利于切除那些直径小或质地柔软的肺小结节。但对于位置过于表浅的微小肺结节以及较深位置的肺结节的定位，这种技术还存在一些不完善的方面。本组病例数较少，在今后的研究中会扩大样本数，改用随机对照研究的方法以获得更为可信的结果。
